# Energetics of whiskered bats in comparison to other bats of the family Vespertilionidae

**DOI:** 10.1242/bio.058640

**Published:** 2021-08-06

**Authors:** Karoline H. Skåra, Claus Bech, Mari Aas Fjelldal, Jeroen van der Kooij, Rune Sørås, Clare Stawski

**Affiliations:** 1 Department of Biology, Norwegian University of Science and Technology, Trondheim NO-7491, Norway; 2 Naturformidling van der Kooij, Rudsteinveien 67, Slattum NO-1480, Norway

**Keywords:** Allometric scaling, BMR, Chiroptera, Insectivorous, *Myotis mystacinus*, Thermoregulation

## Abstract

Bats inhabit a variety of climate types, ranging from tropical to temperate zones, and environmental differences may therefore affect the basal metabolic rate (BMR) of bats from different populations. In the present study, we provide novel data on the energetics of whiskered bats (*Myotis mystacinus*), which is the smallest species within Chiroptera measured to date. We investigated the thermoregulatory strategies of *M. mystacinus* close to the northern limits of this species’ distribution range and compared these data to other vespertilionid bats living in different climates. As mammals living in colder areas experience elevated thermoregulatory costs, often leading to an increase in BMR, we hypothesised that BMR of this northern population of whiskered bats would be higher than that of bats from climates with warm environmental temperatures. From a systematic literature search we obtained BMR estimates (*N*=47) from 24 species within Vespertilionidae. Our metabolic measurements of *M. mystacinus* in Norway (body mass of 4.4 ***g***; BMR of 1.48 ml O_2_ g^−1^ h^−1^) were not different from other vespertilionid bats, based on the allometric equation obtained from the systematic literature search. Further, there was no effect of environmental temperature on BMR within Vespertilionidae. How these tiny bats adapt metabolically to high latitude living is thus still an open question. Bats do have a suite of physiological strategies used to cope with the varying climates which they inhabit, and one possible factor could be that instead of adjusting BMR they could express more torpor.

This article has an associated First Person interview with the first author of the paper.

## Abstract (precis)

**Summary:** The present study provides increased understanding of the energetic costs faced by bats in thermally challenging environments along an environmental temperature gradient.

## INTRODUCTION

The daily energetic challenges experienced by animals readily affect how they function at the individual, population, and species levels (Garland and Adolph, 1991). While energetically expensive, sustaining a high body temperature is often beneficial for endotherms as it allows for behavioural adjustments such as faster movement and the ability to stay active in cold temperatures. All endotherms display a range of temperatures where they do not expend additional energy to maintain a high body temperature, known as the thermoneutral zone. Energy expenditure, or metabolic rate, within the thermoneutral zone is termed basal metabolic rate (BMR) and represents the minimum amount of energy needed to maintain homeostasis (Hill et al., 2016; Withers et al., 2016). As variation in BMR reflects the habitat or ecosystem the animal lives in, BMR is often used as a standard energetic parameter in ecological studies (Garland and Adolph, 1991; White and Kearney, 2013).

Basal metabolic rate varies greatly in mammals, with body mass explaining 95.5% of the overall variation (McNab, 2008; Withers et al., 2016). Body mass thus explains most of the variation in BMR, with the remaining variation being due to a number of factors, such as food habits, climate and habitat (McNab, 2008). It has been suggested that BMR, after removing the effect of body mass, should increase with latitude to meet the energetic demands of inhabiting colder areas (Lovegrove, 2003). An increase in BMR with increasing latitude, and consequently a decreasing environmental temperature, has indeed been found in both mammals (Clarke et al., 2010; Lovegrove, 2003; Raichlen et al., 2010) and birds (Hails, 1983; Tieleman et al., 2002; Weathers, 1979; Williams and Tieleman, 2000). It has also been reported in studies of single mammalian groups, such as canids (Careau et al., 2007) and rodents (MacMillen and Garland, 1989; Rezende et al., 2004; Speakman, 1999). As an increase in BMR with increasing latitude has been found in birds and other mammals, this trend could also be expected for bats. Yet, Cruz-Neto and Jones (2006) and Speakman and Thomas (2003) investigated the effect of latitude on BMR in bats in general, and neither found a significant effect of latitude. As most bat species are found in the tropics (Barclay and Harder, 2003), it has been suggested that environmental temperature could be a better predictor of BMR in bats than latitude (Speakman and Thomas, 2003). However, Willis et al. (2005b) found no effect of mean environmental temperature on BMR among different populations of bats. Nevertheless, heat loss in cold environments is inevitable, and adaptations to inhabit cold environments and living at high latitudes should thus have evolved in bats. As bats inhabit a variety of climate zones, different species and populations may have adapted a variety of metabolic traits to survive the environmental conditions encountered. Belonging to the extremely diverse and species-rich order of Chiroptera, bats are found on all continents except Antarctica, with breeding populations of the northernmost species found at latitudes of 69°N, north of the Arctic circle (Rydell, 1992; Rydell et al., 1994).

Vespertilionidae is the largest family within Chiroptera, making up around one-third of all bat species (Barclay and Harder, 2003). The body size of vespertilionid bats is in general small, varying from 2 to 91 ***g***, and almost all species are insectivorous (Moratelli et al., 2019). The BMR of vespertilionid bats is lower than expected from their body mass compared to other bat species (McNab, 1980b) and to mammals in general, being 53–93% of that predicted for mammals of the same size (Hosken and Withers, 1997). One of the smallest bats within the family Vespertilionidae is the whiskered bat (*Myotis mystacinus*), weighing between 4 and 7 ***g***. The species is observed throughout most of Europe, with the northernmost observation at 64°N (Dietz and Kiefer, 2016; Rydell et al., 1994). Small insectivorous bats at northern latitudes, such as *M. mystacinus*, encounter seasonally cold temperatures and low prey abundance, which impose great energetic costs (Speakman et al., 2000). Due to long winters and short summer nights, the seasonal and daily active periods are limited, which also imposes a constraint on energy acquisition. The thermal challenges associated with these constraints may impact the thermoregulatory curve and BMR of *M. mystacinus*.

Therefore, the aim of the present study was twofold: (1) to measure the BMR and establish the thermoregulatory curve of the previously unstudied *M. mystacinus* inhabiting a seasonally cold environment at their northern distribution limits in Norway, and (2) to investigate whether the BMR of *M. mystacinus* reveal a higher BMR compared to other vespertilionid bats in order to cope with the energetic challenges encountered in a seasonally cold environment. We hypothesised that environmental temperature would affect BMR of *M. mystacinus*, enabling the species to adapt to various climates, including high latitude living. Additionally, we predicted that BMR will be higher in vespertilionid bats living in climates with colder environmental temperatures at high latitudes compared to warmer climates at low latitudes. Knowledge on the thermoregulatory challenges and adaptations encountered by small mammals, such as bats, to a range of environmental conditions is vital for understanding how they may or may not cope with future energetic challenges.

## RESULTS

### Thermoregulation in *M. mystacinus*

**Fig. 1.**
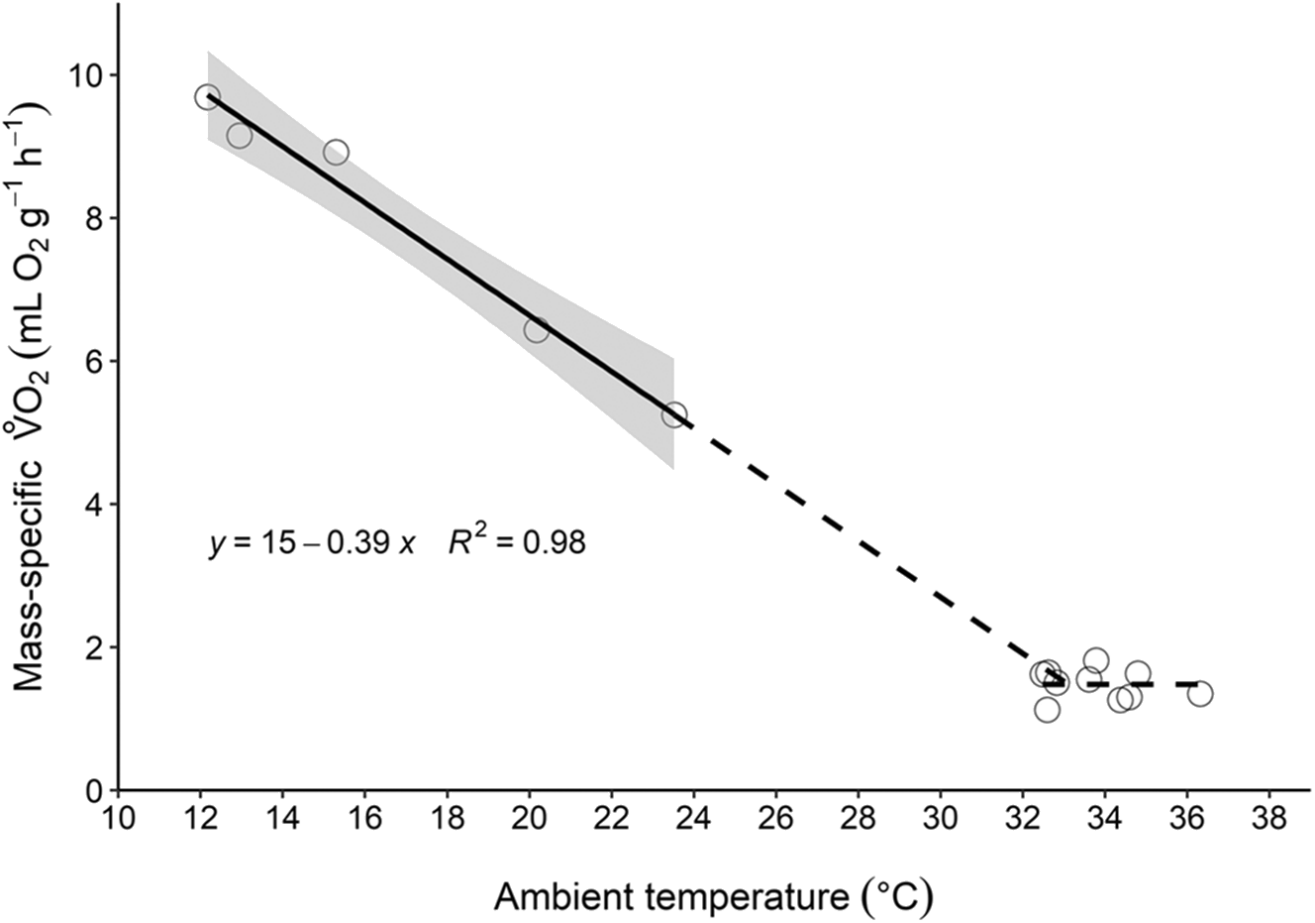
**The thermoregulatory curve obtained for *M. mystacinus*.** The metabolic rate is given as mass-specific 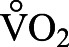 (ml O_2_ g^−1^ h^−1^) as a function of ambient temperature (°C). Each circle represents one 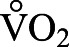 measurement (*N*_individuals_=7, *N*_measurements_=15). The solid line represents the linear regression for resting metabolic rate, with the equation y=14.51–0.39x (*f*_1,3_=162, *P*=0.001, Table S1). The grey area shows the 95% confidence interval for the regression line. The decreasing dashed line is an extrapolation of the linear regression, whereas the horizontal dashed line is a visual representation of the mean BMR.

At low ambient temperatures there was a significant negative effect of ambient temperature on the resting 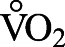 of *M. mystacinus* (*f*_1,3_=162.2, *P*=0.001, Fig. 1 and Table S1), with ambient temperature explaining 98% of the variation in 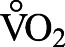. The 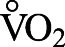 increased by 0.39±0.03 ml O_2_ ***g***^−1^ h^−1^ per 1°C decrease in ambient temperature (body mass=4.9±0.2 ***g***, *N*_individuals_=4, *N*_measurements_=5). The linear relationship between ambient temperature and 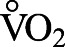 at low ambient temperature is given by the equation y=14.51–0.39x (Table S1). The slope of this regression approximates thermal conductance (0.39 ml O_2_ ***g***^−1^ h^−1^ °C^−1^; body mass=4.9±0.2 ***g***, *N*_individuals_=4, *N*_measurements_=5), and extrapolating the line to the x-axis provided an estimate of body temperature of 37.2°C. The mean BMR was 1.48±0.07 ml O_2_ ***g***^−1^ h^−1^ (body mass=4.4±0.1 ***g***, *N*_individuals_=6, *N*_measurements_=10), illustrated by the horizontal dashed line in Fig. 1. The inflection point between the two dashed lines indicates a lower critical temperature of 33.1°C.

### BMR in Vespertilionidae

**Fig. 2.**
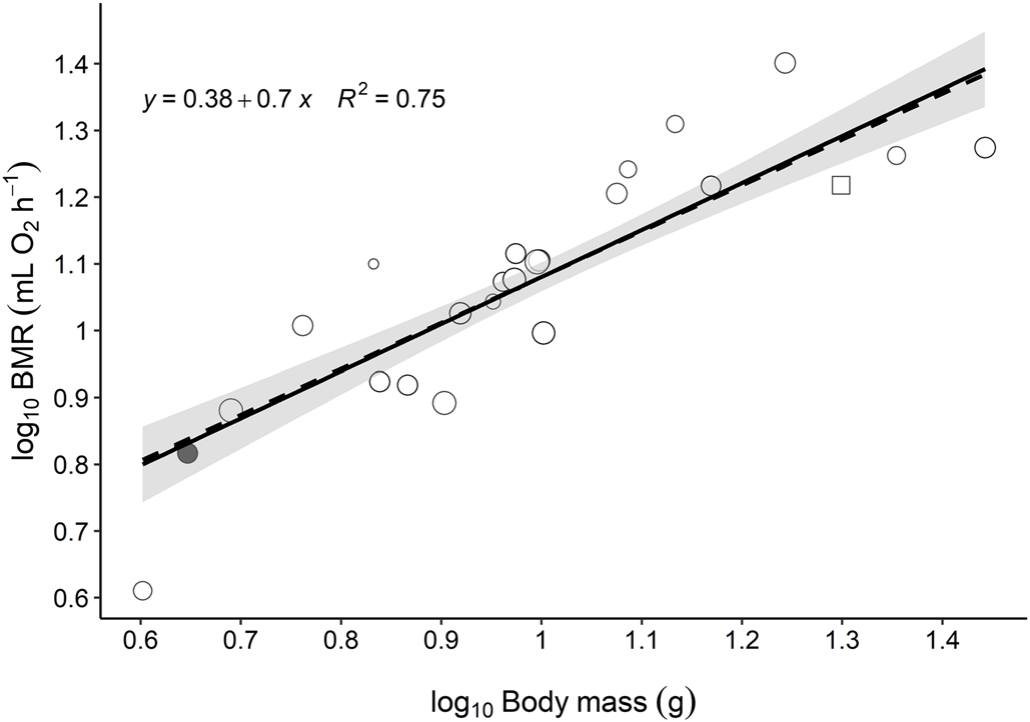
**The relationship between log_10_ body mass (*g*) and log_10_ BMR (ml O_2_ h^−1^) in vespertilionid bats.** The size of the data points represents the precision of the BMR estimate, calculated as the inverse of the standard error (SE) corrected for body mass. The larger the circle, the more precise is the estimate. The solid line and the equation show the linear relationship between body mass and BMR, excluding the BMR estimate of *M. mystacinus* measured in the present study. This estimate is instead illustrated with the filled circle. The non-weighted allometric equation excluding the BMR of *M. mystacinus* was y=0.38+70x (*f*_1,44_=129.0, *P*<0.001, Table S1), whereas including the BMR of *M. mystacinus* it was y=0.37+0.71x (*f*_1,45_=146.6, *P*<0.001, Table S1). The dashed line shows the same relationship as the solid line but calculated using weighted means (y=35+73x, *f*_1,43_=85.2, *P*<0.001, Table S1). The grey area shows the 95% confidence interval for the non-weighted regression. The square shape represents a BMR estimate of one individual, which prevented the calculation of precision.

As expected, the BMR of bats within Vespertilionidae was significantly correlated to body mass (*f*_1,44_=129.0, *P*<0.001, Fig. 2 and Table S1), with body mass explaining 75% of the variation in BMR. This did not include our own BMR measurement of *M. mystacinus*. The equation of the linear relationship between log_10_ body mass and log_10_ BMR was y=0.38+0.70x (Table S1). The slope of this equation, i.e.*,* the allometric scaling exponent, was 0.71±0.01. A weighted linear regression was also calculated, in which each estimate was weighted by their precision. The precision was given as the inverse of the variation, calculated as the mass-specific standard error (SE) (Fig. S4). One of the estimates included in the analysis was a measure of only one individual, and as a result had no measure of variation. This estimate was therefore not included in the weighted regression (dashed line, Fig. 2), but it was included in the non-weighted regression (solid line, Fig. 2) as it did not significantly affect the scaling exponent. There was no significant difference between the weighted and the non-weighted regressions, thus possible variation due to methodology did not affect the results.

**Fig. 3.**
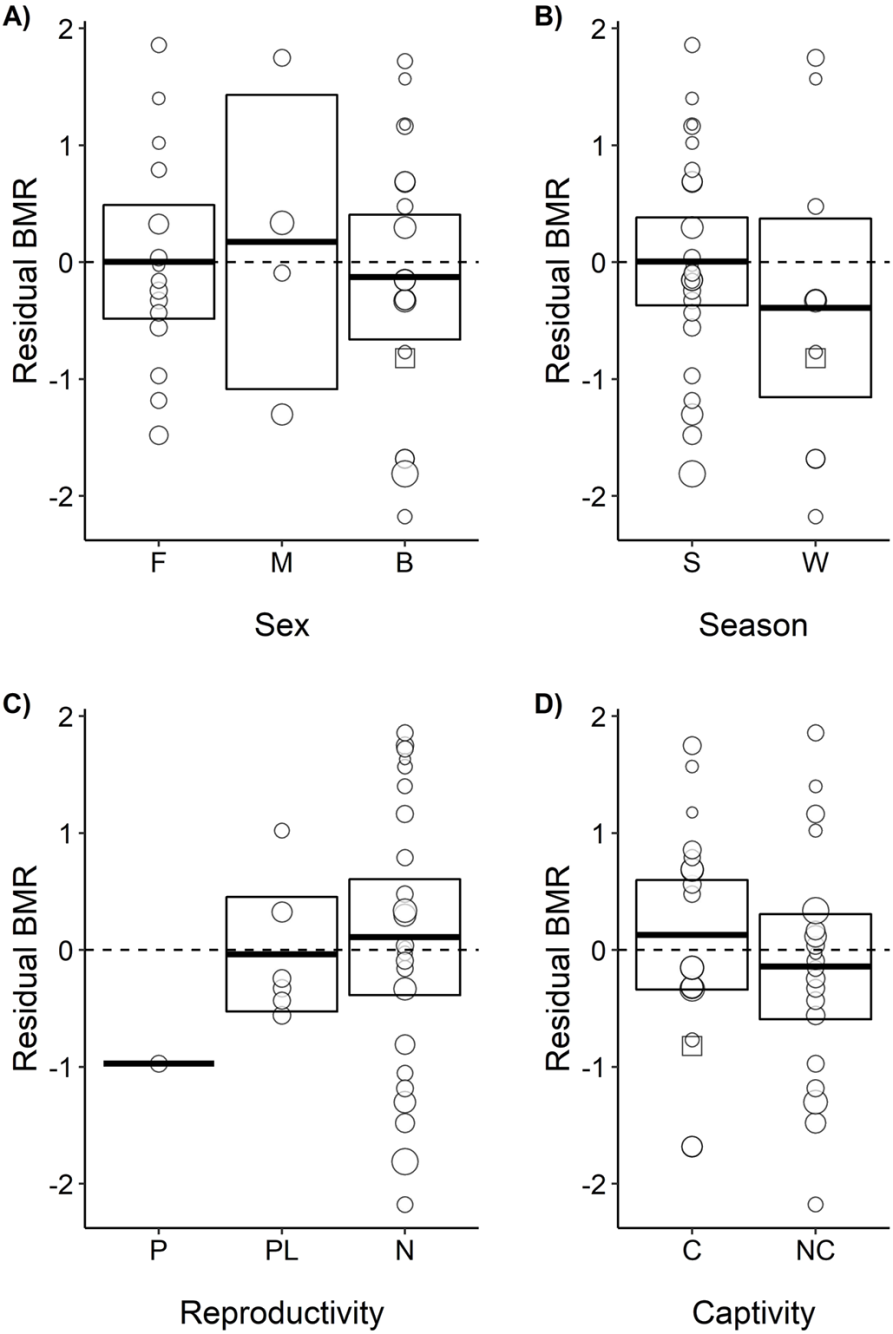
**Residual BMR of bats in different groups.** Effect of sex [females (F), males (M), both (B)], season [summer (S), winter (W)], reproductivity [pregnant (P), post-lactating (PL), non-reproductive (N)] and captivity [captive (C), non-captive (NC)]. Measurements of BMR where this information was not available are not included here but shown in Fig. S1.

The observed BMR for *M. mystacinus* was, based on the data for Vespertilionidae obtained in the present study, 99% of the predicted BMR for a vespertilionid bat of 4.4 ***g*** (Fig. 2). Using the allometric equation for mammals in general from McNab (2008), the mean BMR for vespertilionid bats included in the present study had a BMR 65% of that predicted for a mammal weighing 11.3 ***g***. Using the same equation, the BMR of *M. mystacinus* was 64% of that predicted for a mammal weighing 4.4 ***g***. As body mass had a strong effect on BMR, residuals from the relationship between BMR and body mass were used to compare the BMR independently of body mass. Based on visual inspection, the variance in residual BMR had no obvious relations to differences in sex, time of measurement during the year, reproductive state, or whether kept in captivity or not (Fig. 3). Enough detailed information was not available to test these effects statistically (Fig. S1). Hence, more information is needed to take the possible effects from sex, season, reproductivity and captivity into account.

**Fig. 4.**
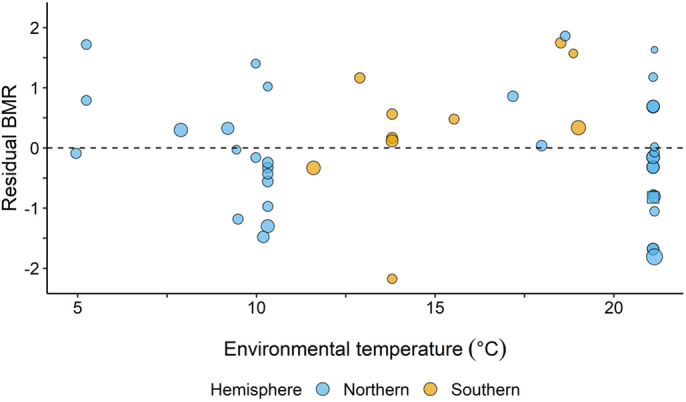
**Residual BMR as a function of mean environmental temperature for species from both the southern and northern hemispheres.** Residual BMR was calculated from the relationship between body mass and BMR from Fig. 2. A residual variation of 0.0 is illustrated by the dashed line. The size of the data points represent the precision of the estimate, calculated as the inverse of the variance corrected for body mass. The larger the circle, the more precise is the estimate. The square shape represents a BMR estimate of one individual, which prevented the calculation of precision.

To analyse the variation in BMR within Vespertilionidae along a temperature gradient, we also included our own BMR estimate of *M. mystacinus* in the allometric equation used to calculate residual BMR. The equation of this allometric relationship was 0.37+0.71x (*f*_1,45_=146.6, *P*<0.001, Table S1). The BMR estimates included in the present study were represented by populations inhabiting areas ranging from 42° S to 60° N (Fig. S2). The measured environmental temperature collected from these locations ranged from a mean of 5.0±0.3°C to a mean of 21.1±1.0°C, representing a wide environmental gradient. However, there was no significant relationship between residual BMR and mean environmental temperature (*f*_1,45_=0.020, *P*=0.66, Fig. 4 and Table S1), even though there was a significant negative relationship between mean environmental temperature and latitude (Fig. S3). The relationship did not change if the southern or northern estimates were excluded.

## DISCUSSION

The present study provides novel data on the thermal energetics of *M. mystacinus* living at high latitudes in Norway, including BMR of the smallest bat species and northernmost bat population ever measured. In addition, we provide information on the variation of BMR within vespertilionid bats along a latitudinal and environmental temperature gradient. Our results contribute to an increased understanding of the energetic costs faced by bats in thermally challenging environments. In contrast to our hypothesis, the population of *M. mystacinus* in the present study revealed a BMR that was not higher than expected from the allometric equation obtained from vespertilionid bats. This was unexpected as a higher BMR was predicted to compensate for increased heat loss to deal with the cold environmental temperature associated with high latitude living. Further, there was no effect of environmental temperature on the BMR in vespertilionid bats that have been studied to date. It is likely that *M. mystacinus* employ other thermoregulatory strategies at their northern range limit to deal with the harsh winter conditions.

The estimated lower critical temperature of 33.1°C for *M. mystacinus* was within the range of what has been measured for other bat species in general, ranging between 25.0 and 34.7°C (Soriano et al., 2002), and also for that of vespertilionid bat species, ranging between 30.5 and 37.7°C (Genoud, 1993; Genoud and Christe, 2011; Kolokotrones et al., 2010; McNab, 2008; Marom et al., 2006; Muñoz-Garcia et al., 2012; Stawski and Geiser, 2011; Willis et al., 2005a,b). In addition, Fristoe et al. (2015) presented data on lower critical temperature of 41 bat species. Using these data, we obtained an allometric equation relating lower critical temperature with body mass within Chiroptera [lower critical temperature (°C)=32.650 × body mass (***g***)^−0.0439^]. As a result of this, we found that the lower critical temperature of *M. mystacinus* was 109% (body mass=4.7±0.2 ***g***, *N*_individuals_=7, *N*_measurements_=15) of the predicted lower critical temperature of bat species within Chiroptera of the same body mass. A steep increase in metabolic heat production was found below this lower critical temperature as ambient temperature declined. This was expected as the body mass of *M. mystacinus* is small, and thermal conductance is generally high for small mammals (Withers et al., 2016). Using the same data from Fristoe et al. (2015) as mentioned above, we obtained an allometric equation relating thermal conductance with body mass within Chiroptera [thermal conductance (ml O_2_ g^−1^ h^−1^ °C^−1^)=0.9307, body mass (***g***)^0.5481^]. Compared to other bat species, the population of *M. mystacinus* measured in the present study had a low thermal conductance, being 88% of that expected for a bat of similar body mass. In addition, the calculated body temperature of 37.2°C for this population was close to a probable body temperature for vespertilionid bats, which has been found to be >32°C in euthermia (Currie et al., 2015; Geiser and Brigham, 2000; Genoud, 1993; Hosken and Withers, 1997, 1999; Withers et al., 2016). Therefore, the low thermal conductance could indicate that this northern population may have evolved good insulation mechanisms to maintain a high body temperature while maintaining the same BMR as bats from warmer climates; thereby avoiding increased energy requirements.

The BMR of *M. mystacinus* was 99% of that predicted for a vespertilionid bat weighing 4.4 ***g***, which indicates that no specific adaptation in BMR has evolved for this specific population to inhabit the cold temperatures at a latitude of 60°N compared to other bat species of Vespertilionidae. Even though the BMR of this population of *M. mystacinus* fitted the allometric relationship well, it is possible that the BMR of southern populations would not as the distribution of this species ranges from 35°N to 64°N (Moratelli et al., 2019). However, no prior studies have investigated the thermal strategies of *M. mystacinus*. Therefore, it remains unknown how the BMR calculated in the present study compares to that of other populations. Importantly, our data are also a vital contribution as no studies have measured the BMR of *M. mystacinus* nor of bats residing at high, northern latitudes, such as in Norway. Using the allometric equation obtained from McNab (2008), the vespertilionid bats from the present study displayed a BMR that was 65%, and *M. mystacinus* a BMR that was 64%, of that predicted for mammals of the same size.

The low BMR in vespertilionid bats compared to other mammals could have evolved to sustain the high energetic requirements encountered by all these species, such as that required by flying or inhabiting thermally challenging environments. In theory, flight by bats increases the metabolic rate 17 times above BMR, so the energetic costs are high, which would also increase body temperature (Withers et al., 2016). Hence, in addition to reducing the overall energetic costs, low BMR could also be a trade-off to avoid increased heat production and overheating during flight. Even though low BMR is characteristic of Vespertilionidae, mass-independent variation in BMR was still present within the family, and this variation remains to be explained.

No effect of environmental temperature on BMR was found in vespertilionid bats, which could indicate that other adaptations to high latitude living have evolved. Bats residing in areas with low environmental temperatures should have to compensate for increased heat loss, for example by revealing higher BMR compared to bats residing in warmer areas. Increasing BMR would enhance cold tolerance, but it would also require even more energy in an already energy scarce environment. If BMR was lowered, on the other hand, it could shift the whole thermoregulatory curve down, reducing the energy requirements even at low temperatures (Stawski et al., 2017; Withers et al., 2016). A decrease in environmental temperature is paralleled by a decrease in available resources, including insects, which could potentially result in a lower BMR in bats inhabiting cold temperatures (Dunbar and Brigham, 2010; Lovegrove, 2000). However, no effect of environmental temperature on BMR was found among the vespertilionid bats included in the present study.

Aside from increasing BMR, there are other potential strategies that bats could employ to deal with the cold. Bats residing in cold environments could increase torpor use, a controlled reduction in metabolic rate and body temperature to reduce thermoregulatory costs, to effectively deal with the cold temperatures. Most bat species studied to date employ torpor, even in warm tropical climates, suggesting it is a vital energy management strategy in bats (Stawski et al., 2014). Interestingly, Cooper and Geiser (2008) found that low BMR was associated with increased use of torpor within rodents, but not within bats. They suggested that bats with high BMR may in fact enter torpor more frequently than bats with low BMR, as their energetic costs are higher and need to be compensated for. Instead, a low BMR could be a result of lower energy availability (Cooper and Geiser, 2008). In addition to the already mentioned strategies, some vespertilionid species are also able to migrate to warmer climates, and escaping the cold may be less energetically costly than coping with it (Fleming and Eby, 2003). Hence, it would be interesting to include information on torpor use and migration patterns in future comparative analyses of BMR among species of Vespertilionidae. However, little detailed information is currently available on these parameters (Moratelli et al., 2019).

The BMR of *M. mystacinus* in Norway, inhabiting a cold environment at a high latitude, was not high compared to other species of bats. In addition, this population did not differ from bats in general in relation to other thermoregulatory parameters, such as lower critical temperature and body temperature. Hence, other adaptations to high latitude living may have evolved for this population. One such adaptation could be increased insulation, for example of fur or fat, resulting in low thermal conductance, as was observed in the present study. Bat species in Vespertilionidae revealed on average low BMR compared to other mammals of the same size. Vespertilionid bats therefore appear to have evolved characteristic traits allowing conservative energy use, such as low BMR and torpor. This is consistent with the high energetic costs associated with for example flying and inhabiting energy-scarce environments. However, as BMR is a trait measured under strict conditions, data selection is an important aspect of a comparative analysis of the variation in BMR. Analysing a poor data set can affect the results considerably, despite including a large sample size. Consequently, data selection should be done with caution (Boyles et al., 2019; Genoud et al., 2018). Energetics studies should include detailed information on methodology and the individuals used to better account for differences between the species included in any future comparative analyses. For bats in particular, the effects of torpor use and roost temperature on BMR are fruitful avenues of research. This is especially interesting to understand how a northern population like *M. mystacinus* is able to cope with the environmental conditions encountered at a high latitude, as no adaptation of BMR was revealed in the present study.

## MATERIALS AND METHODS

Ethics approval to undertake this project was granted by the Norwegian Food Safety Authority (FOTS ID 15944) and the Norwegian Environment Agency (ref. 2018/4899).

### Thermoregulation and BMR in *M. mystacinus*

Male *M. mystacinus* were captured using mist nets during summer 2018 (July to August, *N*=5) and summer 2019 (June to July, *N*=2) in Nittedal, Norway (60°4′23″N, 10°52′20″E), weighing 4.7±0.2 ***g*** (*N*=7) at capture. Bats were transported to a field laboratory immediately after capture, and metabolic measurements were initiated within 2 h after capture. They had no access to food or water before or during measurements and were hence post-absorptive during metabolic measurements. Mealworms and water were provided immediately after the measurements were completed, and the bats were released the following night. Bats were weighed to the nearest 0.1 ***g*** before and after measurements.

Oxygen consumption (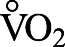, ml O_2_ h^−1^) of the bats was measured using open-flow respirometry. Bats were placed in a respirometry chamber inside a temperature-controlled Pelt Cabinet (Sable Systems International Inc., Las Vegas, NV, USA), which was regulated using a Pelt-5 Temperature Controller (Sable Systems International Inc., Las Vegas, NV, USA). The bats were measured at 10°C until 9 a.m. the morning following capture. The temperature inside the temperature-controlled cabinet was subsequently increased in 5°C increments every hour until the temperature had reached 25°C, after which it was increased in 2°C increments until the temperature had reached 37°C. Each temperature was maintained for at least one hour before increasing to the next temperature, which was enough to ensure that the temperature was stable for at least 30 min.

A FOXBOX analyser (Sable Systems International, Las Vegas, NV, USA) was used for oxygen analysis. The analyser was calibrated using 100% stock nitrogen prior to measurements each year at the university laboratory of Norwegian University of Science and Technology (NTNU) in Trondheim, Norway. During metabolic measurements two pumps were used to push ambient air through two channels, one leading to the respirometry chamber and the other to an empty chamber used for baseline measurements. Both channels led the air through a flow-controller prior to entering the chambers, subsequently through the chambers, and at last to the oxygen analyser. Both the incurrent and excurrent air was scrubbed using silica gel and Drierite prior to entering the chambers and the oxygen analyser. The respirometry chamber which was placed inside the temperature-controlled cabinet had a volume of 325 ml, and hessian fabric was glued to the back of the respirometry chamber allowing bats to hang in a resting position. The oxygen content of air was sampled every minute with a flow rate of 315 ml min^−1^. Baseline measurements of the ambient air were recorded for 15 min at the start of every temperature increase to correct for possible drift in the analyser. The temperature inside the respirometry chamber was recorded with thermocron iButtons (Dallas Semiconductor Inc., Dallas, TX, USA), and the temperature data from these were used in further analyses.

The 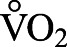 at each set temperature was calculated as the lowest 10-min period of 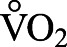 using a 10-min running mean. To ensure that the measurements included in the running mean were obtained from stable 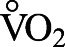 periods, only mean values with an SE below 0.1 ml O_2_ h^−1^ were used. Since the system had a high flow-to-volume ratio, there was no need to use instantaneous 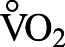 measurements (Bartholomew et al., 1981). The 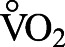 was calculated using Eqn 10.2 from Lighton (2008),
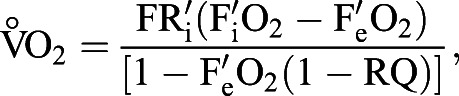
where FR_i_ is the incurrent air flow rate, F_i_O_2_ is the fractional content of incurrent oxygen, F′_e_O_2_ is the fractional content of excurrent oxygen, and RQ is the respiratory quotient, i.e. 
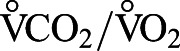
. As 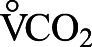 was not measured, a RQ of 0.8 was assumed. As RQ varies from 0.7 to 1.0, using a RQ of 0.8 will produce a relatively small error in 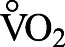, from −3 to 5% (Lighton, 2008). Mass-specific 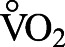 (ml O_2_ g^−1^ h^−1^) was calculated assuming a linear reduction in body mass over time, using the body mass recorded before and after initiation of the metabolic measurements. All 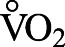 measurements were converted to standard temperature and pressure conditions.

Statistical analyses were performed using the R 3.6.3 software environment (R Core Team, 2020). Metabolic data are given as mass-specific 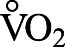 (ml O_2_ g^−1^ h^−1^)±SE. Basal metabolic rate was defined as the mean value of 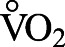 measurements within the thermoneutral zone. However, because *M. mystacinus* is a previously unmeasured species and hence the temperature range for the thermoneutral zone is unknown, the thermoneutral zone was identified based on visual observation and a flattening of the curve at higher ambient temperatures. Resting values of metabolic rate were obtained at ambient temperatures below the flat part of the curve, often referred to as the resting metabolic rate. Using a linear regression of resting metabolic rate against ambient temperature and the calculated BMR, the lower critical temperature was estimated as the inflection point between the two lines. Extrapolation of the linear regression of resting metabolic rate at low ambient temperatures to the x-axis provided an estimate of body temperature, and the slope was considered a measure of the thermal conductance (McNab, 1980a). Raw data are available in Supplementary File 1.

### BMR in Vespertilionidae

A systematic literature search was conducted to obtain data for the comparative analysis of BMR in vespertilionid bats. Inclusion criteria were used to screen papers obtained from the search, whereas exclusion criteria were used to assess the relevant papers for eligibility. The inclusion criteria were (1) a measure of BMR, (2) vespertilionid bats and (3) insectivorous species. The exclusion criteria were (1) a lack of body mass measure and (2) a lack of variation measure for BMR.

The literature was searched with Web of Science using two search terms, one general and one specific. The general search term was used to search through the topic of each paper, i.e.*,* only title and abstract, and contained the following string: “basal metabo*’ OR “BMR” OR “resting metabo*” OR “RMR” AND “bat*” AND “insect*” OR “vespe*” in topic. The specific search term was used to search through entire papers and contained the following string: “basal metabo*” OR “BMR” AND “Bat*” AND “genus” in all fields. The word “Genus” was replaced with each of the 27 genera in Vespertilionidae. This resulted in 27 separate searches using the latter search string. The papers obtained from both the general and the specific search terms were evaluated with the inclusion and exclusion criteria, yielding 17 papers to the comparative analysis.

**Fig. 5.**
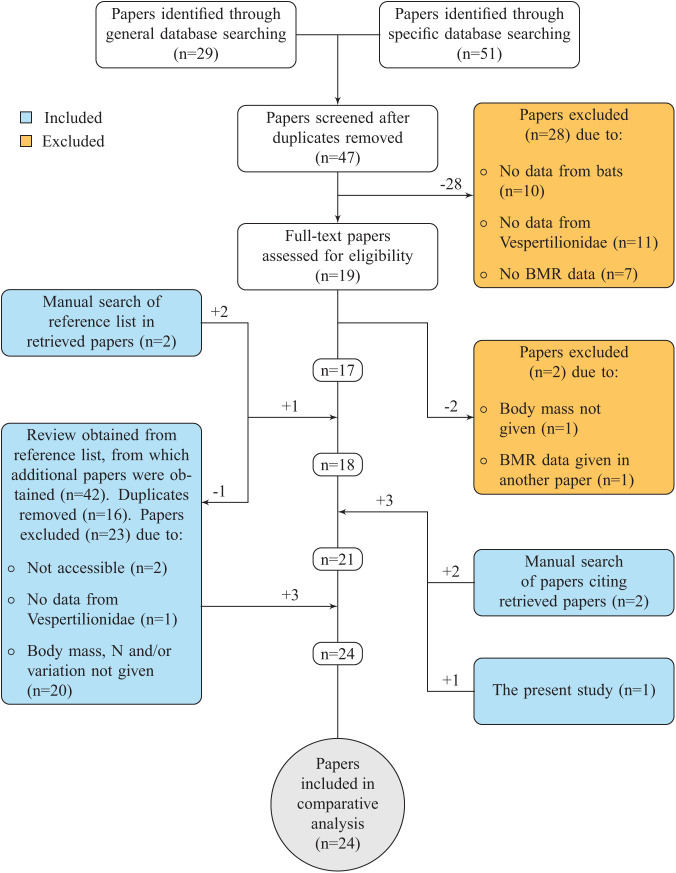
**Flow chart of the systematic literature search.** The search conducted in Web of Science combined inclusion criteria to screen through the abstracts of all relevant papers, and exclusion criteria to carefully evaluate each included paper.

In addition, we went through the reference lists of these 17 papers, and also through the papers that had cited the specific papers. This exercise yielded another six papers with BMR data. Papers were accessed through the electronic collections of the library at NTNU during the period 07.11.2019 to 14.01.2020. A detailed flow chart of the systematic literature search is illustrated in Fig. 5.

The BMR estimate, number of individuals (*N*) used to measure BMR, and the corresponding body mass were extracted from each paper, as well as a measure of variation for both BMR and body mass. Our own respiratory results on *M. mystacinus* were combined with the data from the systematic literature search, creating a final database of 24 species, comprising 47 estimates of BMR. Because more than one estimate of BMR was sometimes retrieved from multiple populations of a single species, we calculated mean BMR for these species. Consequently, this resulted in a total of 24 estimates of BMR used in the statistical analyses. The mean body mass of the bats included was 11.3±0.8 ***g***.

To ensure that the BMR estimates were comparable, the methodology was carefully evaluated in each paper. The BMR estimates that were included were measured using open-flow respirometry in resting, post-absorptive and non-reproductive adults at an ambient temperature within their thermoneutral zone. Where possible based on the information provided in the papers, estimates were classified according to sex, length of captivity, reproductive state, which season the BMR was measured in, and the coordinates where the study was conducted (Fig. 3). However, none of these categories could be used in the quantitative analyses, as all the relevant information was not reported in most papers (Fig. S1). Thus, the categories were not well represented, and were instead used to visualise possible effects graphically.

Measures of BMR were converted to absolute BMR for the analyses, expressed as oxygen consumption per hour (ml O_2_ h^−1^). Estimates given in watts were converted using a conversion factor of 20.1 J ml^−1^, assuming a respiratory quotient of 0.8 (Withers et al., 2016), unless a conversion factor was stated in the original paper. If BMR was given as a median value with range as a measure of variation, it was converted to mean and variance using the method of Hozo et al. (2005). All measures of variation were converted to SE. Body mass and BMR were log-transformed, and standardised residual BMR was calculated from a linear regression of BMR against body mass. Two allometric equations are reported, one equation excluding our own BMR measurement of *M. mystacinus* to compare this estimate to other bats in Vespertilionidae, and one equation including this estimate to analyse the effect of environmental temperature on BMR within Vespertilionidae. All estimates in the comparative analysis are given as means±SE.

Linear models were fitted to the data using the R 3.6.3 software environment (R Core Team, 2020). To determine the effects of mean environmental temperature on BMR between populations of vespertilionid bats, we collected data on environmental temperature from each latitude where BMR measurements were obtained. For the species where we calculated mean BMR based on multiple populations, we also calculated mean environmental temperature. The natural environmental temperature data were obtained from the website World Weather Online (2020), and the means±SE used in the analyses were calculated from January 2009 to December 2019 for the given coordinates obtained from each paper. Data from earlier years were not available. Even though some of the BMR estimates were retrieved from papers older than 2009, a mean of the environmental temperature from 2009 to 2019 was considered representative as a proxy for the different climate types in the given areas. Sufficient information was not provided in the original papers to use seasonal or monthly temperature means, and thus long term mean temperature was considered the best option. Information on latitude was available from most published studies. Where the specific latitude was not reported, we used the location reported in the study to obtain an estimate of the latitude.

**Fig. 6.**
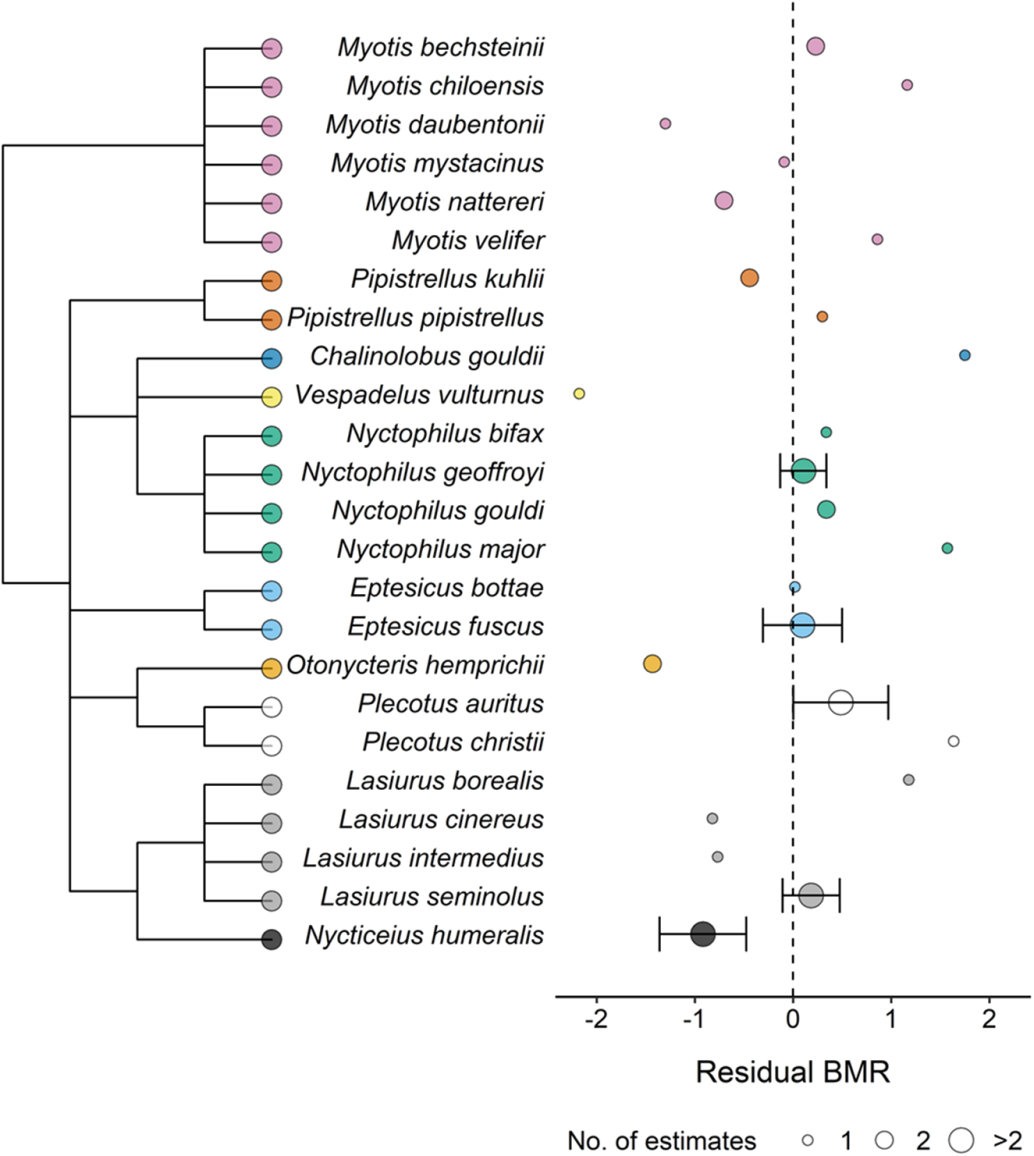
**The residual BMR of the study species included in the comparative analysis.** Residual BMR calculated from the relationship between body mass and BMR in Fig. 2. The phylogeny is shown to the left to visualise the lack of phylogenetic influence on residual BMR. A residual variation of 0 is illustrated by the dashed line. Mean residual BMR was calculated for species with multiple estimates of BMR, shown by increased circle size. Error bars (SE) are presented for means of three or more species.

Multiple environmental factors are found to correlate with BMR in mammals, such as environmental productivity and rainfall parameters (Lovegrove, 2003; Mueller and Diamond, 2001; Withers et al., 2016). It is therefore possible that other environmental factors could be better predictors for BMR than environmental temperature. However, many environmental factors are correlated with each other and we were unable to include more factors due to low sample size and consequently low statistical power. Using latitude information obtained from the studies included in the present analysis, we found that environmental temperature significantly increased with decreasing latitude (Fig. S3). This indicates that environmental temperature captures the environmental variance experienced by vespertilionid bats at different latitudes. Because limited information is available regarding the details of the phylogenetic relationships within Vespertilionidae, we could not obtain a phylogenetic tree of high accuracy. Therefore, we did not statistically test for a phylogenetic signal on BMR among the species. However, a lack of phylogenetic influence on residual BMR is visualised among the species included in the present analysis (Fig. 6).

## Supplementary Material

Supplementary information
