# Supplementary material for: Energetics of whiskered bats in comparison to other bats of the family Vespertilionidae
Source: Biol Open. 2021 Aug 6;10(8):bio058640. doi: 10.1242/bio.058640 (PMC8353265; doi:10.1242/bio.058640)
Supplement: Supplementary information [file biolopen-10-058640-s1.pdf]

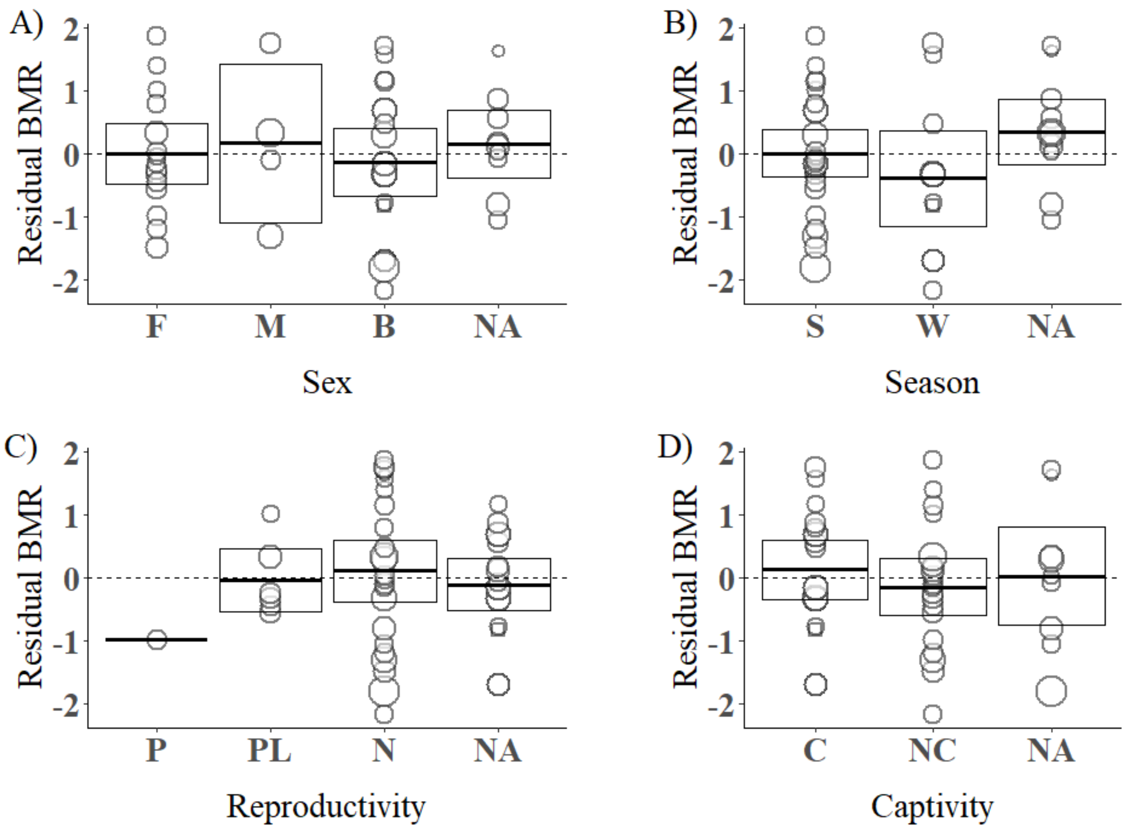

Fig. S1. Residual BMR of bats in different groups. Effect of sex (F=females; M=males; B=both), season (S=summer; W=winter), reproductivity (P=pregnant; PL=post-lactating; N=non-reproductive) and captivity (C=captive; NC=non-captive). Measurements of BMR where this information was not available are also shown (NA=not available).

Biology Open • Supplementary information

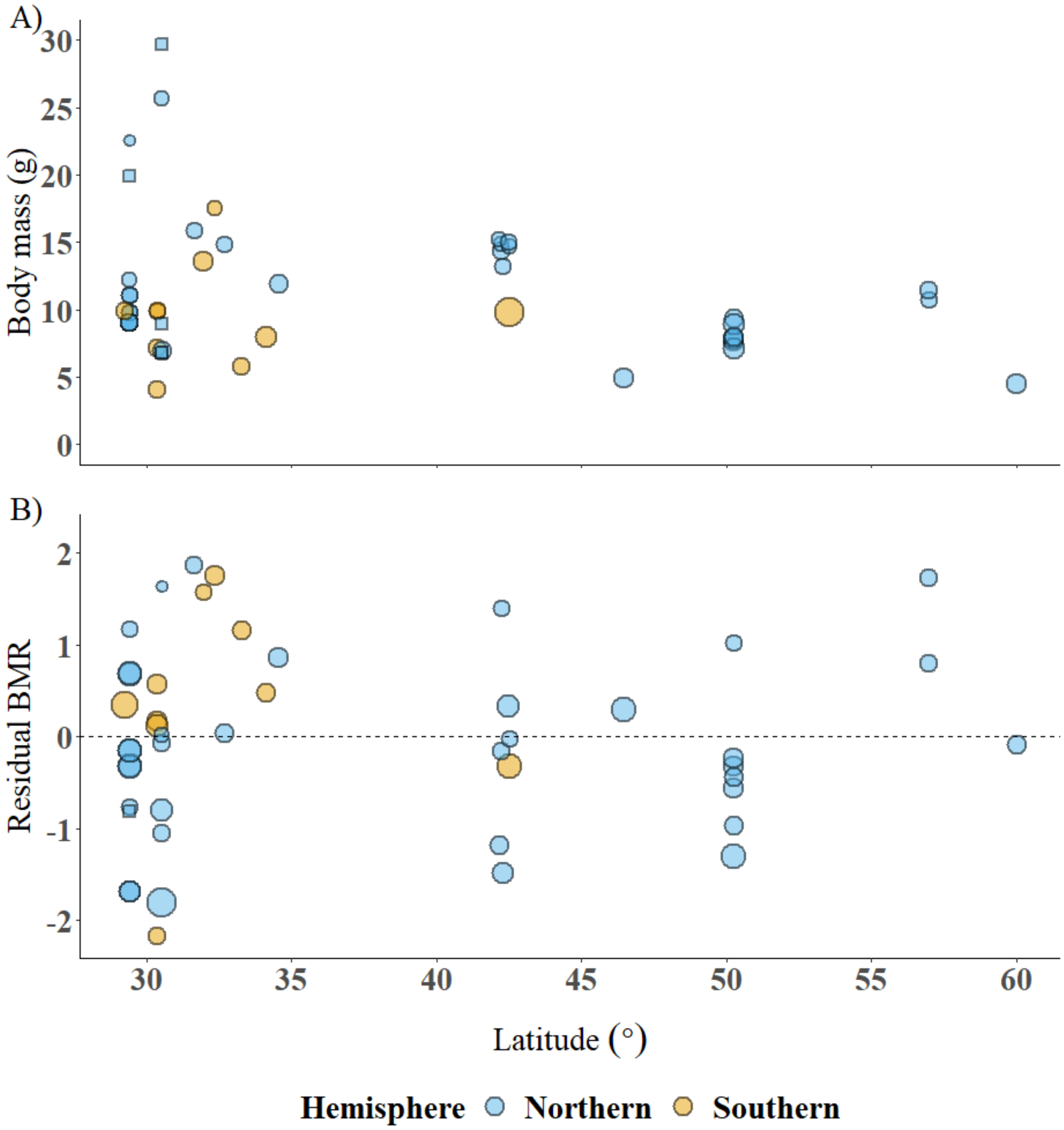

Fig. S2. Body mass (A) and residual BMR (B) as function of latitude. Residual BMR was calculated from the relationship between body mass and BMR in Fig. 3. A residual variation of 0.0 is illustrated by the dashed line. The size of the data point represents the precision of the estimate, calculated as the inverse of the variance corrected for body mass. The larger the circle, the more precise is the estimate. Square shapes represent A) estimates where measures of variation were not obtained, and B) a BMR estimate of one individual, which prevented the calculation of precision.

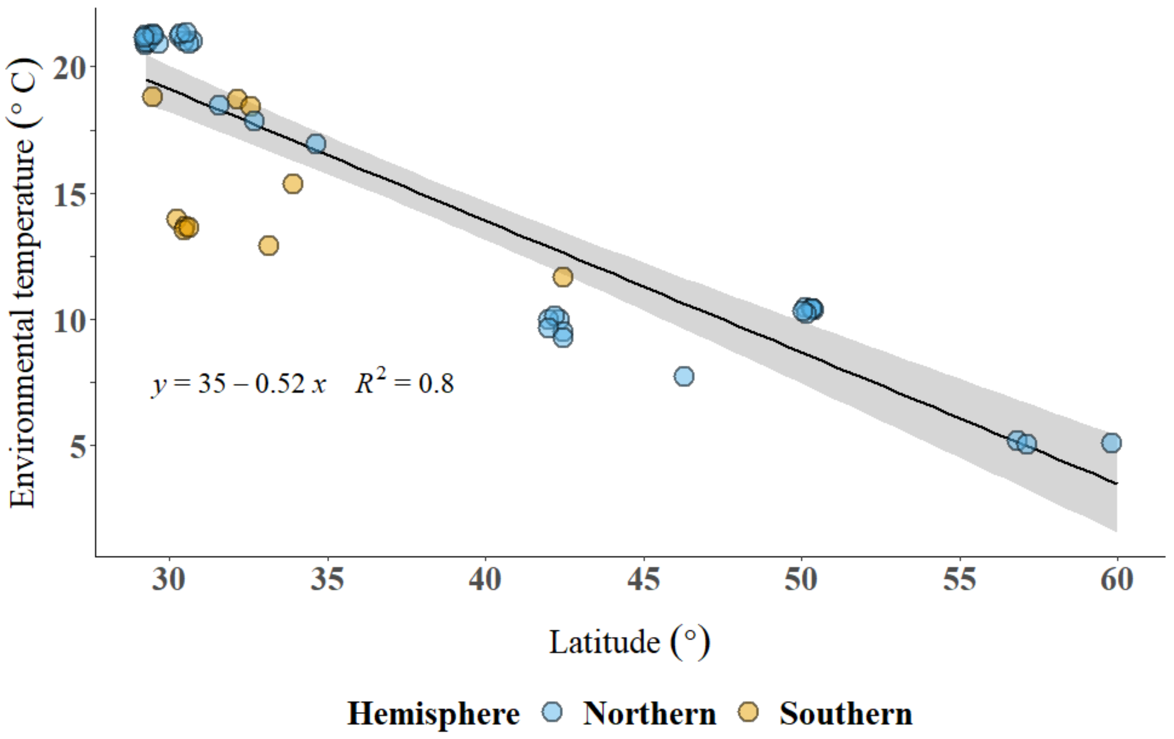

Fig. S3. Average environmental temperature (°C) in the areas of which bats included in the present analysis were measured as a function of latitude (°). The regression equation for this relationship was y = 34.73 – 0.39x ($f_{1,45}$ = 180, $P < 0.001$, Table S1), and the grey area shows the 95% confidence interval for the regression. The temperatures represent yearly average environmental temperature from 2009-2019.

Biology Open • Supplementary information

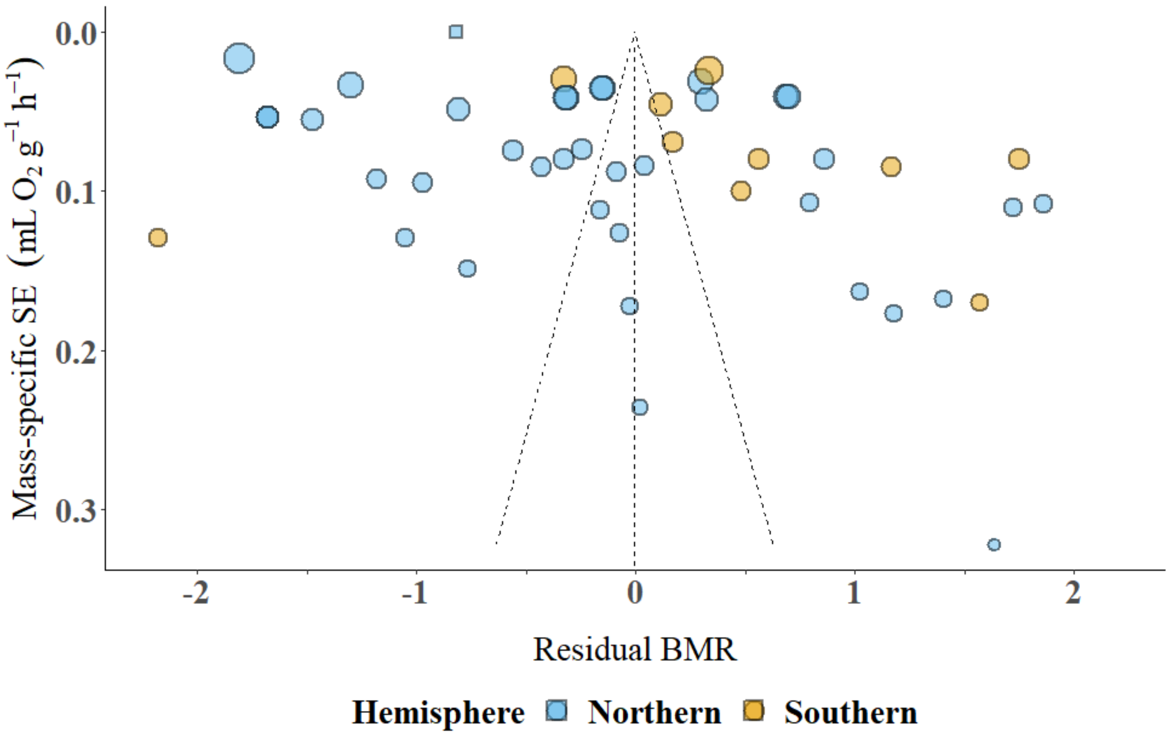

Fig. S4. Mass-specific standard error (SE) (mL O_2 g^{-1} h^{-1}) of residual BMR used to calculate the precision of the included BMR estimates. Estimates with low mass-specific SE, and therefore estimated with high precision, are represented with increased circle size. The mean residual BMR (0.0057 mL O_2 g^{-1} h^{-1}) for the species included in the analysis and 95% confidence intervals are illustrated by the dashed lines.

Biology Open • Supplementary information

Table S1. Summary statistics from the analyses conducted in the present study (SE = standard errors (SE), CI = confidence intervals, N = number of observations, DF = degrees of freedom). Model 1) is from our own metabolic measurements of *Myotis mystacinus*, whereas models 2–6) are from the comparative analysis of BMR within Vespertilionidae.

Coefficient	*Coefficient*	*SE*	*t-value*	*P-value*	*95% CI*
Model 1)	Response: Mass-specific VO_2 (mL O_2 g^{-1} h^{-1})				
Intercept	14.51	0.54	27.02	**<0.001**	[12.80, 16.21]
Temperature	-0.39	0.031	-12.73	**0.001**	[-0.49, -0.30]
N	5				
Residual SE	0.30 on 3 DF				
Multiple R^2 */ adjusted* R^2	0.98 / 0.98				
F-statistics	162.2 on 1 and 3 DF, P-value: **0.001**				
Model 2)	Response: Log BMR, excl. *Myotis mystacinus*				
Intercept	0.38	0.064	5.84	**<0.001**	[0.25, 0.51]
Log body mass	0.70	0.062	11.36	**<0.001**	[0.58, 0.83]
N	46				
Residual SE	0.073 on 44 DF				
Multiple R^2 */ adjusted* R^2	0.75 / 0.74				
F-statistics	129.0 on 1 and 44 DF, P-value: **<0.001**				
Model 3)	Response: Weighted log BMR, excl. *Myotis mystacinus*				
Intercept	0.35	0.081	4.36	**<0.001**	[0.19, 0.52]
Temperature	0.73	0.079	9.22	**<0.001**	[0.57, 0.88]
N	45				
Residual SE	0.091 on 43 DF				
Multiple R^2 */ adjusted* R^2	0.66 / 0.66				
F-statistics	85.1 on 1 and 43 DF, P-value: **<0.001**				
Model 4)	Response: Log BMR, incl. *Myotis mystacinus*				
Intercept	0.37	0.060	6.17	**<0.001**	[0.25, 0.49]
Temperature	0.71	0.059	12.12	**<0.001**	[0.59, 0.83]

N	47				
Residual SE	0.072 on 45 DF				
Multiple R^2 / adjusted R^2	0.77 / 0.76				
F-statistics	146.6 on 1 and 45 DF, P-value: **<0.001**				

Model 5)	Response: Environmental temperature (°C)				
Intercept	34.73	1.50	23.23	**<0.001**	[31.72, 37.74]
Temperature	-0.52	0.039	-13.41	**<0.001**	[-0.60, -0.44]
N	47				
Residual SE	2.48 on 45 DF				
R^2 / R^2 adjusted	0.80 / 0.80				
F-statistics	179.8 on 1 and 45 DF, P-value: **<0.001**				
Model 6)	Response: Residual BMR				
Intercept	0.18	0.45	0.41	0.68	[-0.72, 1.09]
Temperature	-0.012	0.028	-0.45	0.66	[-0.068, 0.043]
N	47				
Residual SE	1.03 on 45 DF				
Multiple R^2 / adjusted R^2	0.0045 / -0.018				
F-statistics	0.20 on 1 and 45 DF, P-value: 0.66				

Table S2

Click here to download Table S2

Biology Open • Supplementary information
